# Impact of social media on triggering nonsuicidal self-injury in adolescents: a comparative ambulatory assessment study

**DOI:** 10.1186/s40479-025-00280-9

**Published:** 2025-01-31

**Authors:** Andreas Goreis, Dorothy Chang, Diana Klinger, Heidi-Elisabeth Zesch, Bettina Pfeffer, Sofia-Marie Oehlke, Ulrich W. Ebner-Priemer, Laurence Claes, Paul L. Plener, Oswald D. Kothgassner

**Affiliations:** 1 Department of Child and Adolescent Psychiatry, Medical University of Vienna, Vienna, Austria; 2 Comprehensive Center for Pediatrics (CCP), Medical University of Vienna, Vienna, Austria; 3 Institute of Sports and Sport Sciences, Karlsruhe Institute of Technology, Karlsruhe, Germany; 4 Department of Psychiatry and Psychotherapy, Central Institute of Mental Health, Heidelberg University, Heidelberg, Germany; 5 Clinical Psychology, Faculty of Psychology and Educational Sciences, KU Leuven, Louvain, Belgium; 6 Faculty of Medicine and Health Sciences, University of Antwerp, Antwerp, Belgium; 7 Department of Child and Adolescent Psychiatry and Psychotherapy, University of Ulm, Ulm, Germany

**Keywords:** Nonsuicidal self-injury, Ambulatory assessment, Social media, Interpersonal stress, Adolescents

## Abstract

**Background:**

Nonsuicidal self-injury (NSSI) is a prevalent and concerning behavior among adolescents, often triggered by negative interpersonal events. As social media is essential in the daily life of adolescents, gaining a better understanding of the impact of negative online events on NSSI urges and behaviors, distinct from that of real-life events, is warranted.

**Methods:**

We recruited 25 adolescents with a history of NSSI and 25 healthy controls. Participants reported on their stress, affect, and NSSI urges four times daily over seven days using ambulatory assessment. We examined the immediate effects of negative events in real-life and on social media on these psychological outcomes.

**Results:**

In adolescents who engage in NSSI, negative events on social media were positively associated with perceived stress, negative affect, and NSSI urges to a greater extent than real-life negative events. However, NSSI events during the sampling period were mostly triggered by real-life events. While the frequency of social media use was generally similar between groups, those with NSSI reported experiencing more negative events on social media.

**Conclusions:**

Our findings highlight the significant impact of social media on the mental health of adolescents who engage in NSSI, possibly exacerbating stress and negative affect more than real-life events. These results underscore the need for targeted interventions addressing online interactions to mitigate NSSI behaviors and improve adolescent mental health.

**Trial registration:**

This study has been registered at the German Clinical Trials Register (ID: DRKS00025905, https://drks.de/search/en/trial/DRKS00025905).

**Supplementary Information:**

The online version contains supplementary material available at 10.1186/s40479-025-00280-9.

## Background

Nonsuicidal self-injury (NSSI) is defined as the deliberate and repetitive infliction of bodily harm without an observable intention to die [31, 35]. NSSI behaviors encompass cutting, scratching, or burning one’s own body tissue, as well as striking or punching objects with sufficient force to cause bruising or bleeding [51]. Global prevalence estimates of NSSI among adolescents range from 16 to 17% [10, 43] up to lifetime prevalence rates of 23% [13], with a peak occurrence between ages 15 and 17, followed by a decrease in young adulthood [38]. Adolescent NSSI has significant repercussions beyond its immediate impact on mental health, including increased healthcare and economic costs, productivity loss, heightened morbidity and mortality [26], and it is a significant predictor of subsequent suicide attempts [5].

Many biological and neurological models [15, 24] posit that NSSI serves to regulate affect and stress (intrapersonal functions). There are, however, also interpersonal social functions, such as influencing the behavior of others or affecting the person’s relationship with others, that are suggested to be relevant underlying functions of NSSI behaviors [6, 17, 36]. Moreover, research indicates significant differences in stress and affective experiences between individuals who engage in NSSI and healthy controls, with the former group often using NSSI as an immediate coping mechanism to alleviate negative emotions [44].

### Intrapersonal vs. interpersonal triggers of NSSI

The question of what precipitates or triggers NSSI acts in the first place, however, has not received as much attention. Studies have shown that negative intrapersonal states, such as perceived stress or negative affect, are higher in those who engage in NSSI [14, 49] and robustly predict NSSI urges, leading to subsequent NSSI acts [23, 50]. This relationship, however, is not as clearly established with regard to interpersonal triggers, such as rejection, social conflict, or criticism. A recent systematic review of interpersonal processes in self-injurious thoughts and behaviors concluded that, overall, studies on the impact of these processes in daily life, as assessed via experience sampling, remain inconclusive [20]. Evidence from qualitative accounts [37﻿], cross-sectional studies [22, 33], and daily life studies [16, 39, 47] supports the notion that interpersonal events, alongside intrapersonal factors such as affect or stress regulation, often trigger NSSI. However, the comparative impact of intra- versus interpersonal triggers has not been directly examined, particularly in adolescents.

### Real-life vs. social media negative events

Another critical aspect that remains insufficiently explored is the distinction between triggering events occurring in real-life and those occurring on social media platforms. Social media is commonly defined as digital platforms facilitating social interaction [34]. These encompass a variety of tools, including social networking sites and apps, such as Facebook, Instagram, and X (formerly Twitter); messaging services such as WhatsApp; online forums and communities like Reddit; and video-sharing platforms such as YouTube and TikTok. The content shared on social media often includes images that can heighten NSSI urges and may lead to self-injurious behavior [4]. Qualitative findings [19, 41] indicate that viewing NSSI-related depictions (e.g., wounds, blood, razors) on social media increases urges, a finding supported by highly controlled laboratory studies [14]. A recent review of 15 studies on the impact of viewing self-harm images on the internet or social media concluded that, while some studies reported positive effects, harmful effects were more prevalent [42]. Social media, therefore, presents a potential intrapersonal trigger, as reading text or viewing pictures can increase negative affect and/or NSSI urges.

As social life and communication also increasingly occur on social media, negative interpersonal events often transpire there [25]. The rapid advancement of technology, coupled with the pervasive use of social media among youth—where these platforms have become central to their interactions and relationships [21]—has raised significant concerns regarding the impact of social media on adolescent mental health [8, 48]. Outside of social media, negative events in daily life have indeed been found to increase negative affect or stress [1, 23], and critically, NSSI urges [47]. However, it remains unclear whether negative events occurring in real-life or on social media have a comparable impact on affect or stress and NSSI urges in the daily lives of individuals who engage in NSSI, particularly among today's youth who are fluent in social media use.

### Present study

The present study aims to elucidate the differential effects of negative (intra- and interpersonal) events in real-life and on social media in the daily lives of adolescents who engage in NSSI and healthy controls. We assessed the characteristics of negative events (i.e., intra- and interpersonal), where they occurred (in real-life or on social media), and their impact on stress, affect, and NSSI urges in adolescents using an ambulatory assessment design over seven days. Self-report data collected in real-time via smartphones multiple times daily through ambulatory assessment [46] has provided valuable insights into the antecedents and consequences of NSSI, primarily in adult samples (see, e.g., [16, 30, 39]). It also allows for examining patterns over time, as previous studies suggest that environmental factors, such as the time of day, may be associated with NSSI acts, urges, and affect, with higher negative affect and increased urges occurring later in the day (e.g., [16]).

It remains unclear whether adolescents engaging in NSSI experience more or fewer daily stressors compared to healthy adolescents, or if both groups are equally affected by real-life and social media stressors. However, based on experience sampling data, adolescents with NSSI exhibit greater affective instability—rapid and intense fluctuations in affect—throughout their days [40]. This increased affective instability possibly leads them to react more intensely, even though the frequency of daily stressors may not differ from that experienced by healthy adolescents. Furthermore, negative affect often precedes NSSI in these individuals [28]. To address this gap, we recruited adolescents who engage in NSSI and a healthy control group to concurrently assess NSSI acts and negative events, enabling a direct comparison of the context and impact of these events across different settings. Specifically, we aimed to address the following research questions:

First, we examined whether the overall within-days trajectories of perceived stress, affect, and NSSI urges in the daily lives of adolescents who engage in NSSI are greater than those of healthy controls. Specifically, we hypothesized that adolescents engaging in NSSI would exhibit higher mean levels of these variables compared to healthy controls over the sampling period. Second, we investigated the triggers of NSSI, aiming to distinguish between intra- and interpersonal events as well as real-life and social media contexts, and to assess their impact on stress, affect, and NSSI urges in participants who engage in NSSI. Third, we explored how and to what extent adolescents who engage in NSSI differ from healthy controls in their responses to negative events in real-life compared to those on social media, focusing on stress, affect, and NSSI urges. In the third research question, we also expected an a priori difference; that is, adolescents with NSSI report a higher impact of negative events—irrespective of their kind—on our outcomes.

## Method

This study was approved by the ethics committee of the Medical University of Vienna (1651/2019). Written consent was obtained from participants and their legal guardian(s) prior to participation.

### Participants

Participants (aged 14–18) were recruited to form two groups: individuals with a history of NSSI or who were currently engaging in NSSI, and healthy controls without a history of lifetime NSSI. The NSSI participants were primarily patients at the Department of Child and Adolescent Psychiatry, Medical University of Vienna, Austria. Healthy controls were recruited from the general public and schools in Vienna and surrounding areas. Recruitment took place from November 2022 to June 2023. The recruitment process utilized multiple methods, including the dissemination of posters, social media advertisements for the healthy control group, and outreach to psychiatric and psychological practitioners in both the private and public health sectors for the NSSI group. To be eligible for the study, participants had to meet specific inclusion criteria: individuals had to be within the age range of 14–18 years. For the NSSI group, participants were required to have a history of at least five NSSI episodes (i.e., self-inflicted damage to body tissue) occurring on at least five separate days within the previous year. Participants who required acute treatment for conditions such as acute psychosis, acute suicidality, or present acute danger of harm to themselves or others were excluded. For the control group, participants were included if they were within the specified age range, reported no lifetime history of NSSI, and had no current or past mental or physical disorders, as confirmed through interviews with both the participants and their caregivers. A total of 30 adolescents with NSSI and 28 healthy controls were screened for inclusion. Of these, *n* = 3 were excluded because they did not respond after initial contact or loss of interest (all in the NSSI group), and *n* = 5 were excluded because their legal guardian(s) did not provide consent for their child's participation (*n* = 2 in the NSSI group, *n* = 3 in the control group). Our trial registration targeted a final sample size of 50 participants, i.e., 25 per group (NSSI and healthy controls), and recruitment was, therefore, halted upon reaching this target. The final sample size of *N* = 50 was selected to accommodate the high number of inpatient cases in our clinical setting and to adhere to Maas and Hox's [32] minimal recommendations for level 2 grouping variables in multilevel ambulatory assessment analyses. All 50 participants completed the study,there was no dropout after inclusion.

### Procedure

Upon inclusion, participants were invited to the Department of Child and Adolescent Psychiatry at the Medical University of Vienna, Austria, for an introductory session. During this session, participants completed baseline measures and were trained in the use of the ambulatory assessment software. The ambulatory assessment software utilized in this study was the movisensXS app (Movisens GmbH). Participants either downloaded the app onto their personal phones or were provided with a study phone for the duration of the study when necessary. Additionally, participants received a study manual containing detailed information regarding the sampling protocol, the meaning of items, and contact details for any questions or technical difficulties.

The ambulatory assessment period commenced the following day and lasted for seven consecutive days. A seven-day period was selected to balance the need for capturing daily fluctuations with the practical considerations of participant compliance to capture a comprehensive and representative sample of their daily experiences. Participants were alerted by an auditory signal to answer questions four times per day: once randomly between 10:00 am and 11:59 am, and three times from 12:00 pm through 8:00 pm, with at least one hour between two subsequent prompts and the possibility to postpone a data entry for a maximum delay of 30 min. The compliance rate for daily assessments was 66%, and did not differ considerably between the groups (NSSI: 65%; control: 67%). In addition to the regular assessments, participants were instructed to report any NSSI acts throughout the assessment period using the same app (event sampling). Upon completion of the study, participants were invited to our laboratories for a post-participation interview and to return the study phones, if provided. Irrespective of response rates, each participant received a €25 voucher as compensation.

## Measures

### Baseline

#### Nonsuicidal self-injury

We used the German version of the revised Self-Injurious Thoughts and Behaviors Interview (SITBI-R, [12]), a validated, semi-structured interview, to assess NSSI presence, frequency, and characteristics.

#### Perceived stress

The German version [27] of the Perceived Stress Scale (PSS-10) by Cohen et al. [7] was used to indicate perceived stress in the last month. For example, participants were asked, "In the last month, how often have you felt that you were unable to control the important things in your life?". All items were rated on a 5-point scale (0 = never, 4 = always). The reliability (McDonald's ω) was 0.91 in the current sample.

#### Depressive symptoms

We employed the German version [29] of the Beck Depression Inventory II [2] to evaluate depressive symptoms experienced in the preceding two weeks. The BDI-II has well-documented validity and reliability in assessing depressive symptoms in children and adolescents. The BDI-II comprises 21 individual questions, each offering four response options on a Likert scale, ranging from 0 to 3. A higher score on a particular item indicates a more pronounced expression of the symptom. The reliability was ω = 0.96 in the current sample.

### Ambulatory assessment

#### Perceived stress

Momentary perceived stress was assessed using a single item, "How stressed do you feel right now?" Participants rated their stress on a visual analog scale (VAS) ranging from 0 (not at all) to 100 (very much). This assessment was conducted four times per day during the ambulatory assessment period.

#### Affect

The 10-item Positive and Negative Affect Schedule (PANAS-SF [45]) was used to assess momentary positive affect (items: active, determined, attentive, inspired, alert) and negative affect (items: afraid, nervous, upset, hostile, ashamed). Participants rated these items on a VAS ranging from 0 (not at all) to 100 (very much) following the assessment of perceived stress. McDonald's ω indicated a generally high reliability in our sample (0.90 for positive affect, 0.94 for negative affect).

#### NSSI Urge

The momentary urge to engage in NSSI was assessed using a single item, "How strong is the urge to harm yourself right now?" Participants rated this on a VAS ranging from 0 (not at all) to 100 (very much) four times per day during the ambulatory assessment period.

#### Negative events on social media and in real-life

Concurrent with each assessment, participants were also asked whether they had used social media since the last assessment. If they responded affirmatively, they were then asked whether a negative event ('something bad') had occurred on social media. If they answered yes, an open-ended response field was provided for them to briefly describe the negative event. Similarly, participants were asked whether a negative event had occurred in real-life. If they responded affirmatively, they were given the opportunity to explain what had occurred in real-life. Negative events were categorized by the ambulatory assessment software into real-life or social media events. Individual text responses were then further classified by the authors, AG and ODK, into interpersonal events—whenever the entry referred to interactions or relationships with another person—and intrapersonal events—whenever the entry referred only to thoughts or activities within the person's own mind.

#### NSSI Acts

After engaging in NSSI and starting the assessment by activating the movisensXS app (i.e., event sampling), participants were presented with several items regarding their NSSI act: the number of wounds/injuries inflicted, how long ago the NSSI act occurred, the duration of the NSSI act, the duration of premeditation before the NSSI act, the methods used, the perceived pain level (rated on a VAS from 0 [not painful at all] to 100 [extremely painful]), and the reasons for engaging in NSSI. All of these items, except for the perceived pain rating, were presented as open-ended response fields. Subsequently, participants who reported NSSI acts were also invited to answer the same stress, affect, and NSSI urge items that were regularly assessed four times per day. They responded to these items immediately after reporting the NSSI acts and were then prompted to respond to these items (stress, affect, and NSSI urge) again at 10, 20, and 30 min following the NSSI acts.

### Analyses

Analyses were conducted via R version 4.1.3, using the packages lme4 and lmerTest to conduct multilevel models, and ggplot2 for data visualization. Plots depicting our data were generated using person-centered means and their standard errors, highlighting both intra-individual variability and inter-group differences. Our research questions were tested via means of χ^2^-tests or using multilevel models to account for variability in both the intercept and slope. In the multilevel models, repeated data entries at level 1 (i.e., stress, affect, NSSI urge, negative events) were nested within participants (level 2).

#### Research question 1

For the comparisons of level 1 outcomes in daily life between NSSI and control participants, (1a) we computed multilevel models with the time-invariant predictor group (factor-coded: NSSI vs. healthy controls) and the ambulatory assessment outcomes (stress, affect, NSSI urge) as dependent variables. Additionally, we included the following predictors as random slopes: (1b) time of day (i.e., assessment time point: morning, afternoon 1 to afternoon 3, factor-coded) and (1c) whether assessments were made on a weekday or during the weekend (factor-coded). Participant ID was modeled with random intercepts and random slopes for time of day and for weekday versus weekend. By allowing covariance among these random effects, we accounted for potential relationships between participants' baseline outcome levels and their trajectories over time.

#### Research question 2

(2a) First, we descriptively analyzed the characteristics of the NSSI behaviors, and (2b) the nature of the triggers of the NSSI behaviors (intrapersonal/interpersonal x real-life/social media). (2c) To analyze the immediate effect—and the short-term trajectories—of triggers of NSSI on stress, affect and NSSI urge, we computed multilevel models with the variable time as predictor (last assessment before the NSSI acts, immediately after, and + 10, + 20, and + 30 min after; factor-coded) and the four outcomes (i.e., stress, positive and negative affect, NSSI urge) as dependent variables. Only participants in the NSSI group and those who reported having engaged in NSSI during the assessment period (n = 12) were included in this analysis. Similar to research question 1, Participant ID was modeled as a random intercept and time as a random slope.

#### Research question 3

We first compared both groups (NSSI vs. healthy controls) regarding (3a) the frequency of social media use, (3b) the nature of negative events (intrapersonal vs. interpersonal) on social media and in real-life they encountered using χ^2^-tests. (3c) To analyze the impact of negative (intra/interpersonal) events on social media and in real-life on our outcomes (stress, affect and NSSI urge), we computed models with the interaction event type (real-life vs. social media) x negative event occurred since the last assessment (yes/no) and our four outcomes. Participant ID was again modeled as a random intercept and negative event occurred as a random slope. These models were computed twice—once for the NSSI group and once for healthy controls. Significant interactions were followed up with Tukey’s post-hoc tests.

## Results

The final sample consisted of fifty participants: 25 who engaged in NSSI (*M*_age_ = 15.88, 72% female) and 25 healthy controls (*M*_age_ = 16.56, 68% female), all self-identified as White. The groups were comparable in terms of age and gender. On average, participants in the NSSI group engaged in NSSI 1.16 times in the past week, 5.28 times in the past month, and 69.4 times in the past year. The most common NSSI methods were cutting (80%, *n* = 20), hitting themselves or objects (28%, *n* = 7), burning (20%, *n* = 5), scraping or scratching (28%, *n* = 7), biting (8%, *n* = 2), and inserting objects under the skin (8%, *n* = 2). Additionally, the NSSI group reported higher perceived stress and more depressive symptoms than the control group (both *p*s < 0.001). Within the NSSI group, 64% had a preexisting mood disorder, 48% had personality disorders, and 60% were on psychopharmacological medication (Table 1).

**Table 1:** Participant characteristics

Group	NSSI (*n* = 25)	Control (*n* = 25)
	*M (SD)*	*M (SD)*
Age	15.88 (1.17)	16.56 (1.66)
Gender		
Female	18 (72%)	17 (68%)
Male	2 (8%)	8 (32%)
Diverse	5 (20%)	0 (0%)
NSSI 1-week prevalence (SITBI-R)	1.16 (1.82)	–
NSSI 4-week prevalence (SITBI-R)	5.28 (7.1)	–
NSSI 1-year prevalence (SITBI-R)	69.4 (62.69)	–
Perceived Stress (PSS-10)	27.32 (6.76)	16.20 (7.34)
Depressive Symptoms (BDI-II)	31.96 (13.52)	8.40 (8.05)
Previous Diagnoses (ICD-10)		
F3x Mood [affective] disorders	16 (64%)	–
F4x Neurotic, Stress-Related and Somatoform Disorders	7 (28%)	–
F5x Behavioural Syndromes Associated with Physiological Disturbances and Physical Factors	1 (4%)	–
F6x Disorders of Personality and Behaviour	12 (48%)	–
F9x Behavioural and Emotional Disorders	8 (32%)	–
Current Psychopharmacological Medication	15 (60%)	–

### Research question 1. Comparison of daily-life stress, affect, and NSSI urge between the two groups

**Fig. 1:**
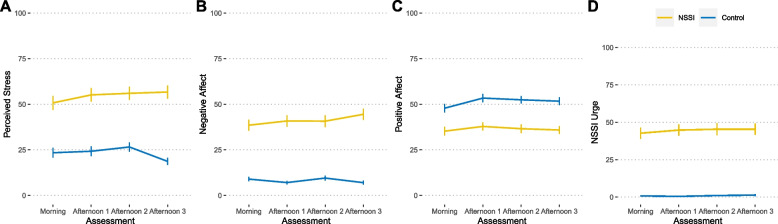
Within-day trajectories of perceived stress (**A**), negative affect (**B**), positive affect (**C**), and urge to engage in NSSI (**D**) for both groups over the four assessment time points (*M* ± *SEM*)

As depicted in Fig. 1, our analyses indicated that the NSSI group consistently exhibited higher levels of perceived stress (*b* = 30.78, *p* < 0.001), negative affect (*b* = 27.85, *p* < 0.001), and urges to engage in NSSI (*b* = 38.26, *p* < 0.001) in their daily lives compared to the control group. Additionally, the NSSI group demonstrated lower levels of positive affect (*b* = −15.25, *p* = 0.002). The time of day did not significantly affect stress (*p*s > 0.138), positive affect (*p*s > 0.695), or NSSI urges (*p*s > 0.457).

However, time of day was significantly associated with negative affect, particularly during the final measurement of each day (see Fig. 1, "Afternoon 3" timepoint) which showed that the NSSI group—but not the control group—experienced higher negative affect values at the end of each day (interaction group x time of day: *b* = 4.99, *p* = 0.027). On the contrary, weekday or weekend did not affect stress (main effects: *b* = −3.03, *p* = 0.393), positive affect (*b* = 1.07, *p* = 0.592), negative affect (*b* = −1.32, *p* = 0.574), or NSSI urge (*b* = −2.57, *p* = 0.322). Characteristics (*M, SD,* and range) and intraclass correlation of all ambulatory assessment variables are reported in Supplementary Table 1.

### Research question 2. Characteristics of NSSI, triggers of NSSI, and consequences of NSSI engagement on stress, affect and NSSI urges in Daily Life

#### Characteristics of NSSI

In total, 21 NSSI acts were reported. Twelve participants in the NSSI group reported engaging in NSSI during the study period. The primary method of NSSI was cutting (13 events), followed by carving (7 events). The time span with the highest occurrence of NSSI acts was between 4 and 8 PM, with 9 events recorded during this period. The duration of premeditation before engaging in NSSI varied: less than a minute (29% of events), 1–15 min (also 29%), or 16–60 min (33%). Perceived pain associated with NSSI acts was rated as *M* = 53.45 (*SD* = 25.96) on a scale from 0 to 100. Supplementary Table 2 provides a detailed list of all NSSI acts, their reasons, and methods.

#### Triggers of NSSI

Participants attributed their NSSI to interpersonal social conflicts (13 NSSI acts, e.g., fights with a partner), followed by intrapersonal (stress-related) reasons (7 NSSI acts, e.g., inability to handle pressure, feeling bad). For one NSSI act, no reason was given by the participant. Only one interpersonal trigger reportedly occurred on social media (receiving bad messages on social media), whereas zero intrapersonal triggers were associated with social media. Therefore, 18 out of 20 NSSI acts were attributed to real-life triggers.

#### Effect of NSSI acts on stress, affect and NSSI urges

**Fig. 2:**
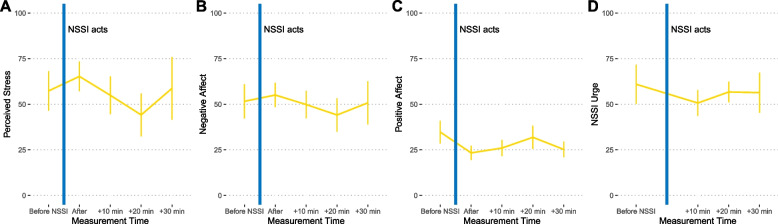
Perceived stress (**A**), negative affect (**B**), positive affect (**C**), and urge to engage in NSSI (**D**) before and after NSSI acts (*M* ± *SEM*)

We initially planned to analyze triggers of NSSI acts separately based on intra- and interpersonal events as well as real-life and social media contexts. However, due to the uneven distribution of reported triggers, this approach was unfeasible. Therefore, we analyzed all NSSI acts collectively, regardless of trigger category. After examining the short-term trajectories of stress, affect, and NSSI urges (see Fig. 2) that occurred before and after NSSI acts, our analyses revealed that engaging in NSSI was not significantly associated with any outcomes, i.e., there were no significant changes from before to after engaging in NSSI (stress: *p* = 0.750, negative affect: *p* = 0.556, positive affect: *p* = 0.507, NSSI urge: *p* = 0.371). These four analyses were based on 21 data points collected before the NSSI events (as there were 21 NSSI events overall) and a total of 262 (out of 336 possible) follow-up data points for the four outcomes. This means that 22% of event-contingent follow-ups after NSSI events were not recorded by participants and were therefore missing.

### Research question 3a. Comparison of social media use between groups

During the 7-day assessment, participants reported using social media at 66% of all assessment times, with no significant difference between the usage rates of both groups (NSSI group: 67%, control group: 64%, χ^2^ = 0.89, *p* = 0.345).

### Research Question 3b. Frequency of intrapersonal/interpersonal negative events on social media and in daily life between groups

Participants in the NSSI group reported more negative events on social media (13 events, or 5% of all social media uses) compared to the control group (2 events, or 1%, χ^2^ = 9.42, *p* = 0.002). Among the negative events on social media in the NSSI group, 11 were social in nature (7 occurred in group settings, e.g., mobbing or exclusion in group chats or social media posts/comments, and 4 occurred in one-on-one communications, e.g., with a partner or strangers). Two negative events were intrapersonal in nature and related to consuming NSSI content online that participant described as triggering.

The two negative events on social media reported in the control group included one social, interpersonal event (a fight with a group of friends) and one intrapersonal event related to distressing news consumption about global conflicts.

Regarding real-life negative events, the NSSI group reported significantly more incidents (32 events, or 9% of all assessments) than did the control group (12 events, or 3%, χ^2^ = 13.00, *p* < 0.001). In the NSSI group, these 32 negative real-life events consisted of 24 social interactions (17 in group fights/ostracism contexts and 7 in one-on-one interactions) and 8 intrapersonal or work-related stressors (e.g., work/school stress, victim of burglary, and gender dysphoria due to suboptimal medical procedures). In the control group, the 12 negative real-life events included 7 interpersonal social interactions (4 in group fight/ostracism contexts and 3 in one-on-one interactions) and 5 intrapersonal/work-related stressors. See Supplementary Tables 3 and 4 for a complete list of reported negative events on social media and in real-life.

### Research Question 3c. Impact of the presence/absence of real-life and social media negative events on stress, affect and NSSI urges

**Fig. 3:**
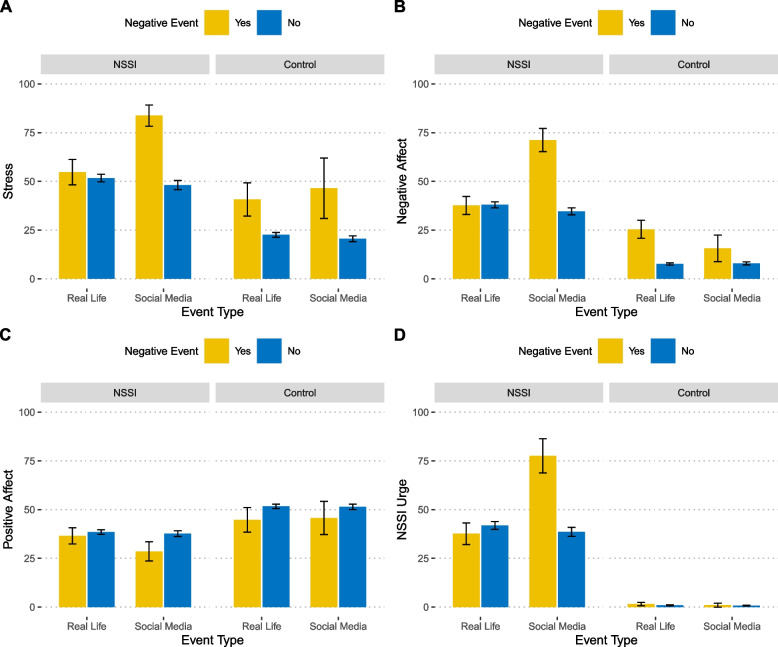
Impact of the occurrence/non-occurrence of negative events, the presence of real-life and social media negative events and their interactions on Perceived Stress (**A**), Negative Affect (**B**), Positive Affect (**C**), Urge to Engage in NSSI (**D**) in the NSSI and control group (*M* ± *SEM*)

Next, we analyzed whether reporting a negative event concurrently affected our outcomes (stress, affect, NSSI urge) and whether this was influenced by the nature of the negative events (i.e., real-life vs. social media). In the NSSI group, we found significant interactions of event type (real-life/social media) x event type (occurred/not occurred) for the outcomes stress (*p* = 0.005), negative affect (*p* = 0.005), and NSSI urge (*p* = 0.008). Post-hoc tests subsequently revealed a relatively coherent pattern of associations: the presence of negative events on social media was associated with significant increases in stress, negative affect, and NSSI urges compared with when they did not (Tukey's post hoc tests: all *p*s < 0.031), and the increases in stress, negative affect, and NSSI urges were always greater in the case of negative social media events than in the case of negative real-life events (Tukey's *p*s < 0.044; see Fig. 3 for a graphical depiction of comparisons). In the NSSI group, positive affect was generally lower when negative events occurred than when negative events did not occur (main effect: *p* = 0.022), but positive affect was not influenced by the event type (real-life/social media (main effect of event type: *p* = 0.119). Finally, the interaction of occurrence x type was not significant (interaction: *p* = 0.094).

In the control group, significant main effects of the occurrence (vs. non-occurrence of) were found on stress (*p* = 0.029) and negative affect (*p* = 0.036), indicating that any negative event was associated with increased perceived stress as well as greater negative affect. No significant main effect was found for occurrence on the outcome positive affect (*p* = 0.668). Due to the small base rate of negative social media events in the control group, we refrained from conducting statistical analyses of event type or any interactions in this group. However, for transparency, we reported outcomes descriptively (see Fig. 3), where NSSI urges of the healthy control group are also depicted but not analyzed.

## Discussion

The present study investigated the differential effects of negative (intrapersonal/interpersonal) events in real-life and on social media on stress, affect, and NSSI urges in adolescents who engage in NSSI and healthy controls. Using an ambulatory assessment design, we explored an array of psychological outcomes and how they were affected by negative events directly in the daily lives of adolescents. Our findings indicate that negative events occurring on social media significantly exacerbate perceived stress, negative affect, and NSSI urges more than do negative events occurring in real-life among adolescents who engage in NSSI. Conversely, this was not the case in our sample of healthy controls, where those adolescents reported a negative—albeit lower than the NSSI group's—impact of either negative event, whether on social media or in real life, on stress and negative affect. Our findings, thus, underscore the potent influence of social media on the mental health and behavior of adolescents who engage in NSSI.

Consistent with other studies, we found in Research Question 1 that being in the NSSI group—compared with the control group—was associated with heightened stress, higher negative affect, and lower positive affect, even without considering negative events of any kind [49, 50]. Both stress and negative affect (such as feelings of hopelessness, guilt, and rejection) are frequently reported to be linked with NSSI engagement [18]. Therefore, our direct comparisons between affective states in adolescents with NSSI and healthy controls confirm this notion and underscore the psychological distress reported by individuals who engage in NSSI.

With respect to actual NSSI acts, studies have found that people who engage in NSSI find it difficult to explicitly recognize interpersonal motives for engagement in NSSI [3], even in ambulatory assessment designs [39]. We, however, found in Research Question 2 that two-thirds of NSSI acts were attributed to interpersonal conflict. One possibility for this difference from other studies (which report relatively fewer social triggers and more intrapersonal ones, [16]) is that participants in our study were given the opportunity to write their reasons in open-ended response fields (and not choose from a pre-defined list), potentially elaborating on their motives. Furthermore, all but one NSSI act was attributed to real-life events. One possible explanation for this discrepancy is that negative events on social media may increase NSSI urges by elevating stress and negative affect, whereas actual NSSI behaviors may be more directly triggered by immediate and severe real-life stressors. Thus, online negative events could influence internal emotional states, while tangible real-life stressors might prompt self-injurious actions. However, on the basis of the low prevalence of NSSI acts in our study, this remains speculative and requires further research. A majority of everyday life studies in this field also rely on adult populations (which are less socially active on social media than adolescents are), which may contribute to the higher prevalence of social functions of NSSI engagement in adolescents.

To our knowledge, this is the first study directly assessing the effects of negative events on social media (compared to real-life negative events) on stress, affect and NSSI urges in adolescents with NSSI (Research Question 3). Generally, it is assumed that youth with a history of NSSI are more susceptible to the adverse effects of negative social media interactions [4, 9], which can exacerbate emotional distress and NSSI urges and even NSSI behaviors. Furthermore, viewing self-harm images can negatively impact NSSI behavior [42]. We found that adolescents with NSSI and healthy controls used social media with very similar frequency (during 67% and 64% of all assessments, respectively) but those with NSSI reported more negative events while using social media. It is noteworthy that internet or social media use can also be used positively to connect and support each other in online NSSI groups [11]. For the purpose of our study, we focused (and thus instructed participants) only to report negative events on social media and may therefore have missed additional and potentially positive functions of internet/social media use. Negative events in real-life were also more frequent for the NSSI group, with nearly three times as many reported. Due to the relatively low number of occurrences on social media, we were, unfortunately, unable to directly compare the impact of intra- and interpersonal functions and may only give an overview of the overall impact. However, we note that more than two-thirds of all reported negative events—whether in real life or on social media—were of an interpersonal nature. Interpersonal events are, therefore, very often a trigger for NSSI urges and lead to them in line with previous findings [16]—a finding that we were overall able to corroborate. Overall, we found that potentially triggering negative social media events have a more pronounced impact on stress and negative affect than real-life events in adolescents who engaged in NSSI compared to healthy controls.

### Limitations and future directions

This study has several limitations that should be considered. First, the sample size, while adequate for detecting significant effects and similar to other daily life studies in this area of research [39], was relatively small, and the findings may not generalize to all people or even all adolescents who engage in NSSI. Second, NSSI acts were not that common in our sample, which is similar to other daily life studies in this area (see [16]). Third, most analyses were contemporaneous, preventing us from establishing the directionality or causality between negative events and NSSI urges. Consequently, NSSI urges may have prompted adolescents to seek out NSSI-related content on social media, or increased social withdrawal during high NSSI urges may have led to greater exposure to negative online events, rather than negative events causing the urges. Additionally, the potential for alpha error accumulation should be acknowledged, as multiple statistical tests increase the risk of Type I errors. On a similar note, future research could replicate our findings regarding the differential impact of social media and real-life triggering factors, and actually compare inter- and intrapersonal events. Future investigations should also aim to include more diverse samples to improve the generalizability of the results. Our adolescents in the NSSI group were primarily in mental health care and, therefore, might not fully represent all adolescents who engage in NSSI. Lastly, the reliance on self-reported data for both negative events and emotional states may introduce bias. Incorporating objective measures, such as physiological indicators of stress, could provide a more comprehensive understanding of the impact of negative events on NSSI behaviors.

## Conclusion

In conclusion, this study provides novel insights into the differential impacts of negative events in real-life and on social media on adolescents who engage in NSSI and healthy controls. Our findings highlight the heightened vulnerability of adolescents who engage in NSSI to social media-induced stress and underscore the importance of addressing online interactions in both clinical and policy contexts. Given the significant role of social media in adolescents' lives, these findings have several practical implications for mental health interventions and policies. Clinicians should incorporate discussions of social media use into their therapeutic work with adolescents who engage in NSSI and talk about strategies to manage their reactions to negative online interactions and limit their exposure to triggering content, which could be crucial components of effective NSSI interventions. Furthermore, we echo recommendations that social networks implement stringent content moderation protocols [4] and that educational institutions and community organizations implement programs to increase awareness of the impact of ostracism and exclusion in digital environments.

## Supplementary Information

Supplementary Material 1.

## Data Availability

The datasets used and/or analyzed during the current study are available from the corresponding author on reasonable request.

## Abbreviations

NSSI: Nonsuicidal self-injury
SITBI: Self-Injurious Thoughts and Behaviors Interview
PSS: Perceived Stress Scale
BDI II: Beck Depression Inventory II
VAS: Visual Analog Scale
PANAS: Positive and Negative Affect Schedule
