# Supplementary material for: Impact of social media on triggering nonsuicidal self-injury in adolescents: a comparative ambulatory assessment study
Source: Borderline Personal Disord Emot Dysregul. 2025 Jan 31;12:4. doi: 10.1186/s40479-025-00280-9 (PMC11786379; doi:10.1186/s40479-025-00280-9)
Supplement: Supplementary file 1 — Supplementary Material 1. [file 40479_2025_280_MOESM1_ESM.docx]

**Appendix**

**Supplementary Table 1. Characteristics and Intraclass Correlation Coefficients (ICCs) of Ambulatory Assessment Variables.**

| Group | NSSI | Control | ICC |
| --- | --- | --- | --- |
|  | *M (SD)* [range] | *M (SD)* [range] |  |
| Perceived Stress | 54.92 (35.85) [0–100] | 23.21 (24.81) [0–100] | .613 |
| Negative Affect | 42.37 (29.64) [0–99] | 8.13 (11.75) [0–70.2] | .800 |
| Positive Affect | 35.16 (21.52) [0–91.4] | 51.45 (21.97) [1.2–100] | .558 |
| NSSI Urge | 45.44 (35.01) [0–100] | 0.92 (4.64) [0–70] | .791 |

**Supplementary Table 2. List of NSSI Events.**

| **ID** | **Reason NSSI Event** | **NSSI Method** | **Painful (0–100)** |
| --- | --- | --- | --- |
| 1 | Bad mood at school, feeling alone, friend not there. | Scratching | 57 |
| 1 | Classmate's suicide | Burning, Scratching | 77 |
| 2 | Friend treated me badly | Cutting | 74 |
| 2 | Partner treated me badly | Cutting (Hand) | 75 |
| 6 | Relationship issues | Carving | 0 |
| 8 | Couldn't handle it anymore | Cutting | 71 |
| 8 | Received negative message on social media | Cutting | 62 |
| 11 | – | Cutting | 86 |
| 33 | Life feels overwhelming | Carving | 36 |
| 33 | Can't take it anymore | Cutting | 64 |
| 33 | Because of my partner | (Using a) Pencil | 42 |
| 34 | Flashback from trauma | Cutting | 38 |
| 34 | Flashback from trauma, did not have enough from first time [i.e., first NSSI event] | Cutting | 36 |
| 36 | Angry at myself | Carving | 3 |
| 39 | Feeling pressured | Carving | 32 |
| 39 | Can't handle sadness | Cutting with a Knife | 75 |
| 39 | Bullying and friends | Carving | 72 |
| 39 | Conflict with friend | Cutting | 66 |
| 43 | Feeling bad | Cutting | 10 |
| 46 | Thinking of ex and memories | Cutting | 71 |
| 47 | Can't handle jealousy | Carving + Cutting | 75 |

**Supplementary Table 3. List of Social Media Negative Events.**

| **ID** | **Group** | **Negative Event (Social Media)** | **Category** |
| --- | --- | --- | --- |
| 12 | Control | Argued with friends | Interpersonal/social |
| 3 | Control | The news is distressing and affects me deeply (Qatar, Iran, Ukraine etc.) | Intrapersonal/distressing news/war |
| 46 | NSSI | Memories of ex were triggered | Interpersonal/social |
| 45 | NSSI | Group chat was mean and triggered me | Interpersonal/social |
| 43 | NSSI | Saw pictures that almost triggered me | Intrapersonal/NSSI content |
| 43 | NSSI | Memories resurfaced through private message | Interpersonal/social |
| 43 | NSSI | Pictures almost triggered me | Intrapersonal/NSSI content |
| 42 | NSSI | Hate comments | Interpersonal/social |
| 40 | NSSI | Saw triggering pictures on a friend's account that reminded me of my trauma | Intrapersonal/NSSI content |
| 39 | NSSI | Argument in group chat, wasn't invited again | Interpersonal/social |
| 39 | NSSI | Argued with friends, bullying | Interpersonal/social |
| 39 | NSSI | Was kicked out of group chat | Interpersonal/social |
| 11 | NSSI | Someone from school keeps texting me saying she's bored. I'm not her entertainment and I don't like being used for that. | Interpersonal/social |
| 2 | NSSI | Argument with partner | Interpersonal/social |
| 45 | NSSI | Was disinvited from a party on WhatsApp | Interpersonal/social |

**Supplementary Table 4. List of real-life negative events.**

| **ID** | **Group** | **Negative Event (Real-Life)** | **Category** |
| --- | --- | --- | --- |
| 24 | Control | My parents told me that if I didn't go to their university of choice across the world, they would actively work to deport me. | Interpersonal/social |
| 18 | Control | Parents argued | Interpersonal/social |
| 17 | Control | Had an argument with my brother that escalated quickly | Interpersonal/social |
| 12 | Control | Dad prefers someone else at the moment | Interpersonal/social |
| 4 | Control | My 17-year-old friend is pregnant and we don't know what to do | Interpersonal/social |
| 4 | Control | Stubbed my toe | Intrapersonal/work stress |
| 3 | Control | A customer was demanding | Intrapersonal/work stress |
| 24 | Control | My mom said a bunch of triggering stuff I couldn't handle | Interpersonal/social |
| 23 | Control | Bad grade | Intrapersonal/work stress |
| 20 | Control | Official termination | Intrapersonal/work stress |
| 18 | Control | My mom yelled at me because I couldn't find my Christmas money, which was last in her bag | Interpersonal/social |
| 9 | Control | Lost a volleyball match (by a large margin) | Intrapersonal/work stress |
| 47 | NSSI | Argument with friend | Interpersonal/social |
| 45 | NSSI | Arrived late to school | Intrapersonal/work stress |
| 43 | NSSI | Argument in the shared apartment, with tears | Interpersonal/social |
| 43 | NSSI | Continued arguing with friends | Interpersonal/social |
| 42 | NSSI | People said things to me that were just not okay | Interpersonal/social |
| 42 | NSSI | Argued with friends | Interpersonal/social |
| 38 | NSSI | Theft | Intrapersonal/work stress |
| 36 | NSSI | Insults from coworkers | Interpersonal/social |
| 36 | NSSI | Was insulted by a stranger | Interpersonal/social |
| 33 | NSSI | Argument with my boyfriend | Interpersonal/social |
| 16 | NSSI | Sister, father, and I argued | Interpersonal/social |
| 15 | NSSI | Was excluded and left behind by a group | Interpersonal/social |
| 8 | NSSI | Had an outing planned but my mom is sick, no one else can take over except a friend, which is not possible | Interpersonal/social |
| 8 | NSSI | My mom lost her phone | Intrapersonal/work stress |
| 7 | NSSI | Did some sports after a long time, realized how much I've declined, which stressed me out | Intrapersonal/work stress |
| 7 | NSSI | A harsh reminder of a part-time job I neglected and the associated guilt, feeling I exploited someone's trust | Intrapersonal/work stress |
| 7 | NSSI | Unexpected encounter with a mentally ill person that triggered me | Interpersonal/social |
| 6 | NSSI | Had a date and waited 1-2 hours but they didn't show up | Interpersonal/social |
| 6 | NSSI | A confrontation I don't know how to handle | Interpersonal/social |
| 2 | NSSI | Not a big deal, but my stepmother was a bit angry with me | Interpersonal/social |
| 2 | NSSI | My brother yelled at me about something he gave me but forgot about. Not a big deal because he is mentally ill | Interpersonal/social |
| 1 | NSSI | Since Friday, when I mentioned what happened, I haven't felt good. My concentration, attention, and patience are completely gone. I feel only emptiness inside | Interpersonal/social |
| 1 | NSSI | A boy from the neighboring class committed suicide | Interpersonal/social |
| 1 | NSSI | I mentioned a friend's suicide in group therapy and it apparently upset others, making me feel very guilty | Interpersonal/social |
| 45 | NSSI | Disinvited from a party | Interpersonal/social |
| 43 | NSSI | Work stress, feeling overwhelmed | Intrapersonal/work stress |
| 38 | NSSI | Impulsive behavior from relatives | Interpersonal/social |
| 34 | NSSI | I got my period even though my endocrinologist prescribed the pill, and I haven't had it since then. Gives me gender dysphoria | Intrapersonal/work stress |
| 16 | NSSI | Argued with sister, but reconciled | Interpersonal/social |
| 15 | NSSI | Afraid my friend will leave me, intense self-hatred | Interpersonal/social |
| 15 | NSSI | A stressful conversation about my health, I wasn't taken seriously | Interpersonal/social |
| 11 | NSSI | Realized I can't study properly for the test because the teacher doesn't explain why certain steps are taken | Intrapersonal/work stress |
